# Patient Perceptions of Orthopedic Surgeon-Led Nutrition Discussions Regarding Arthroplasty Care: A Single-Center Retrospective Study

**DOI:** 10.7759/cureus.98116

**Published:** 2025-11-29

**Authors:** Janae Rasmussen, Linda Rasmussen, Elise Rivera, Cody Walter

**Affiliations:** 1 Orthopedic Surgery, Valley Consortium for Medical Education, Modesto, USA; 2 Orthopedic Surgery, Windward Orthopaedic Group, Kailua, USA; 3 Osteopathic Medicine, A.T. Still University School of Osteopathic Medicine, Mesa, USA

**Keywords:** arthroplasty surgeon, hip & knee arthroplasty, nutrition education, orthopedic practice, total joint arthroplasty

## Abstract

Background: Nutrition and lifestyle factors, such as low albumin, smoking, and morbid obesity, have been linked to worse outcomes after arthroplasty surgery. This study is the first, to our knowledge, to investigate the effect of orthopedic surgeon-led nutrition discussions on patient perceptions in arthroplasty care. The primary objective of this study was to describe patient perceptions and self-reported experiences regarding nutrition counseling provided directly by their orthopedic surgeon in the context of arthroplasty care. Secondary objectives were to assess the following: (1) patient recall of receiving nutrition guidance; (2) the perceived benefit of this counseling on their surgical preparation and recovery; and (3) patient attitudes toward surgeon-led nutrition discussions.

Methods: In this retrospective study, patients who underwent hip, knee, or both hip and knee arthroplasty between 2004 and 2024 at a single-center private practice orthopedic group were surveyed regarding nutrition counseling. A chart review assessed for complications, comorbidities, and surgical outcomes. A subgroup analysis was performed to evaluate whether patient age differed between those who reported adopting a low-carbohydrate diet based on their surgeon's guidance and those who did not. Mean ages were compared using a Welch two-sample t-test with significance set at p < 0.05. Additional categorical subgroup analyses were performed to determine the difference in infection status based on smoking status (Fisher's exact test), age (Welch two-sample t-test), and alcohol use (Cochran-Armitage trend test). Importantly, this study is unable to analyze associations with complications due to an insufficient event rate. Note that several of the optional questions only had 146 responses, so those questions were not included in the statistical analysis.

Results: A total of 152 patients responded to the survey (not all questions had responses), representing a total of 190 primary arthroplasty procedures and three revisions. Of these, 114 patients (78.1%) reported they wanted their orthopedic surgeon to discuss nutrition, 17 (11.6%) were unsure, and 15 (10.3%) did not. Most patients (n = 127; 87%) indicated that surgeon-led nutrition counseling positively impacted their care, while 12 (8.2%) were unsure, and seven (4.8%) reported no benefit.

Conclusion: This study demonstrated patient-perceived benefits of surgeon-led nutrition counseling in arthroplasty care. These findings highlight the potential value of incorporating nutrition discussions into the surgical care pathway. However, this study is not able to assess objective clinical benefits due to limitations in the study design. This study only provides preliminary, hypothesis-generating data on patient attitudes, not evidence of efficacy or benefit. Given the single-center, retrospective design and limitations of this study, further multicenter investigations are warranted to assess outcomes and inform standardized guidelines.

## Introduction

The role of nutrition in optimizing recovery in joint arthroplasty has increasing relevance due to the literature highlighting nutrition's impact on outcomes [1-3]. Adequate nutrition is critical for muscle preservation, wound healing, and overall postoperative outcomes [3]. Prior literature has demonstrated the importance of optimizing nutrition in orthopedic patients, with markers such as albumin influencing wound healing and recovery [4]. Dubé et al. explored the impact of preoperative malnutrition and how it is associated with worse outcomes following total joint arthroplasty, including increased infection rates, prolonged hospital stays, higher rates of reoperation, and elevated mortality [5]. They highlighted the challenges of assessing nutritional status in orthopedic patients, noting the reliance on biomarkers, such as serum albumin, prealbumin, transferrin, and total lymphocyte count [5]. Additional large-scale studies demonstrate that malnutrition is strongly correlated with adverse arthroplasty outcomes. Cross et al. emphasized that hypoalbuminemia and malnutrition independently predict complications in orthopedic surgery, including infection, delayed healing, and higher readmission rates [6]. Similarly, Fu et al. reported that hypoalbuminemia is a stronger predictor of complications than obesity following total knee arthroplasty (TKA) [7]. 

Assessing a patient's nutritional status can often be overlooked in patients with obesity, despite malnutrition being prevalent in this population [8]. There are no consistent body mass index (BMI) cutoffs for total joint arthroplasty as it is surgeon-dependent, which emphasizes the value of orthopedic surgeons' understanding of nutrition's role when discussing surgical options with arthroplasty candidates [4,9]. 

There is also a lack of standardized screening methods for nutrition in arthroplasty surgery, which limits the consistency of preoperative evaluations. Importantly, a patient's nutrition is related to social determinants of health and health literacy, such as their ability to understand nutrition labels, which emphasizes the need for a more holistic approach to patient risk stratification in arthroplasty [5]. Low health literacy has been linked to worse health outcomes across multiple settings, underscoring the importance of integrating nutrition education with broader patient education strategies [10]. While nutrition is an important factor in perioperative management, it may be inadequately addressed in orthopedic surgery. 

Despite substantial evidence on the importance of nutrition in osseous and wound healing in orthopedic surgery, there remain underdeveloped standardized nutritional strategies for arthroplasty patients. Enhanced Recovery After Surgery (ERAS) protocols have been widely adopted in orthopedic procedures, particularly total joint arthroplasty, to improve patient outcomes through a multifaceted approach [11]. These protocols emphasize preoperative optimization, including patient education and early mobilization [11]. The most recent ERAS Society consensus guidelines specifically highlight perioperative nutrition screening and intervention as essential elements of arthroplasty recovery, though they acknowledge gaps in standardized implementation [12]. While ERAS protocols recognize the importance of nutrition, perioperative counseling and intervention guidelines in arthroplasty remain under-investigated. It is unclear whether patients undergoing arthroplasty surgery receive sufficient preoperative and postoperative nutrition guidance. It is also unclear what nutrition recommendations should be provided, which may limit an orthopedic surgeon's comfort in counseling their arthroplasty patients on nutrition.

In this study, a single arthroplasty surgeon focused on nutrition education and counseling on dietary modifications, specifically a high-protein and low-carbohydrate diet, for their arthroplasty patients, a practice maintained for over 20 years. This study did not investigate the validity of specific dietary recommendations as the focus was on patient perceptions of nutrition counseling. While the impact of nutritional status on objective surgical outcomes is well-documented, there is a paucity of data on how patients perceive nutrition discussions when they are initiated directly by the orthopedic surgeon. Therefore, this study aimed to assess patient-reported perceptions and subjective experiences in patients who had undergone joint arthroplasty with preoperative nutrition counseling provided directly by their treating orthopedic surgeon. We hypothesized that the majority of patients would report a positive perception of nutrition counseling by their orthopedic surgeon.

The primary objective of this study was to describe patient perceptions and self-reported experiences regarding nutrition counseling provided directly by their orthopedic surgeon in the context of arthroplasty care. Secondary objectives were to assess the following: (1) patient recall of receiving nutrition guidance; (2) the perceived benefit of this counseling on their surgical preparation and recovery; and (3) patient attitudes toward surgeon-led nutrition discussions.

## Materials and methods

Study design and participants

This study surveyed eligible patients treated between 2004 and 2024 using a 33-item questionnaire created in Google Forms (Google, Mountain View, CA, USA) and was conducted at Windward Orthopaedic Group in O'ahu, Hawai'i. This 20-year retrospective period is a notable source of potential recall bias. The survey was distributed through a QR code printed on mailed letters and clinic rooms, which may have introduced selection bias, especially for individuals with low technological literacy. The total number of surveys distributed was not tracked due to limitations in assessing whether clinic fliers were taken or the QR code was scanned, which also limits the understanding of the response rate. There were no follow-up reminders. Inclusion criteria were patients aged 18 years or older who had undergone partial knee arthroplasty (PKA), TKA, or total hip arthroplasty (THA) performed by the single treating surgeon in private practice.

A total of 152 individuals initiated the survey. Of these, 146 participants completed all required questionnaire items and were included in the primary analytic sample. Birthdates were reviewed solely for the purpose of calculating participant age. Eleven respondents entered birth years that were implausible or could not be interpreted (e.g., including values outside a physiologically possible adult range or future years). These entries could not be converted into ages and were excluded only from age-specific analyses while being retained in all other analyses. This resulted in 135 participants with interpretable age data.

The survey assessed patient perceptions of nutrition discussions with their orthopedic surgeon and self-reported quality of life. A chart review was conducted to evaluate postoperative complications and to capture medical and surgical histories, which were not blinded.

Survey development and piloting

Since no validated survey instruments were available on this topic, survey items were developed by two authors (JR, CW) and reviewed by the senior author (LR). The survey was piloted among several patients to ensure readability and comprehension, with minor edits made before launch. The full 33-item survey is provided in the Appendices.

Chart review

Three reviewers (JR, LR, ER) extracted surgical history (e.g., procedure type, laterality, date, index vs. revision, operating surgeon/site) and correlated the data with survey responses. For patients with a documented infection, reviewers captured details including infection type (e.g., periprosthetic joint infection (PJI) vs. superficial), timing relative to index surgery, diagnostic evidence, and management approach (e.g., antibiotics vs. two-stage revision). Reviewers were not blinded to survey responses due to workflow constraints. No discrepancies in the chart review were identified.

Data management

Survey responses were exported from Google Forms into Google Sheets for initial review and organization. Data were checked manually for completeness and consistent labeling (e.g., "low-carb" and "low-carbohydrate") before analysis. "Unsure" responses were retained rather than reported as missing. The cleaned dataset was then imported into R Version 4.2.3 (R Foundation for Statistical Computing, Vienna, Austria) for descriptive and inferential analyses. No data imputation or transformation beyond these steps was performed.

Statistical analysis

Age, smoking, and alcohol use were selected based on clinical plausibility and prior associations with arthroplasty outcomes, though they were performed post hoc and interpreted as exploratory. Subgroup analyses were performed to evaluate whether patient age differed between those who reported adopting a low-carbohydrate diet based on their surgeon's guidance and those who did not. Mean ages were compared using a Welch two-sample t-test with significance set at p < 0.05. Given the exploratory design, p-values are descriptive without multiplicity correction. Complication analyses used Fisher's exact tests for binary exposures (e.g., smoking yes/no) and the Cochran-Armitage trend test for ordered alcohol-use categories. Age differences by complication status used Welch's t-test. To enable reproducibility under small counts (total complications n = 6), exact cell sizes for each test are reported. Distributional assumptions were not tested. Findings should therefore be interpreted as hypothesis-generating.

Additional categorical subgroup analyses were performed to assess the difference in infection status based on smoking status (Fisher's exact test), age (Welch two-sample t-test), and alcohol use (Cochran-Armitage trend test). 

The survey addressed domains related to perioperative nutrition counseling and patient-reported experiences. No power calculation was performed due to the retrospective design. Optional items with missing data were excluded pairwise with the denominator reported per item; "Unsure" was analyzed as its own category. Statistical analysis was conducted in R Version 4.2.3, with survey data collected via Google Forms and organized in Google Sheets as described above. This study's limitations restrict statistical interpretations.

## Results

A total of 152 patient survey responses were included, representing 190 primary arthroplasty procedures and three revisions: 50 THA, 123 TKA, and 17 PKA (Table 1). Participants ranged in age from 41 to 90 years (mean 70 years). The average BMI was 30.7 (range 17.8-49.6). Gender distribution was as follows: 59% women, 41% men, and 0% other/prefer not to say. Age distribution by decade is reported and summarized here: 41-50 (n = 2), 51-60 (n = 3), 61-70 (n = 52), 71-80 (n = 60), 81-90 (n = 17), and 90-99 (n = 1). 

**Table 1:** Arthroplasty surgeries reported by patient survey participants and verified by chart review. THA: total hip arthroplasty; TKA: total knee arthroplasty; PKA: partial knee arthroplasty

Surgery	Number of surgeries	
Left THA	25	Total THA: 50
Right THA	25	
Left TKA	52	Total TKA: 123
Right TKA	71	
Left PKA	8	Total PKA: 17
Right PKA	9	
Right PKA revision	1	Total revisions: 3
Right TKA revision	1	
Left TKA revision	1	
	Total: 193	

Medical history is detailed in Table 2.

**Table 2:** Medical history of the participants. A chart review was performed to verify any listed medical history of the survey participants. HTN: hypertension; HLD: hypercholesterolemia; BMI: body mass index; CAD: coronary artery disease; CKD: chronic kidney disease; NAFLD: non-alcoholic fatty liver disease; IBD: inflammatory bowel disease; COPD: chronic obstructive pulmonary disease

Medical history of the participants		Number of patients
Prior nicotine use		49
Gastric bypass		8
Type 1 diabetes mellitus		0
Type 2 diabetes mellitus		26
HTN		78
HLD		46
Obesity (BMI not specified)		50
Rheumatoid arthritis		6
Psoriasis		2
CAD		11
CKD		3
NAFLD		3
Gout		8
IBD		1
Asthma		23
COPD		6
Sleep apnea		34
Depression		12
Anxiety		9
Thyroid disease		23
Peripheral arterial disease		3
Stroke		4
Alcohol use	None	52
	Rarely	54
	1-2 drinks per week	13
	3-5 drinks per week	20
	6+ drinks per week	8

Six complications were identified (Table 3): one transient peroneal nerve palsy, one superficial infection (cellulitis) treated with antibiotics, one allergic reaction to closure adhesive, and three PJIs requiring two-stage revision. Notably, one PJI occurred after an index surgery performed elsewhere, and one patient was on chemotherapy at the time of infection.

**Table 3:** Summary of complications of patient survey participants using chart review. BMI: body mass index; THA: total hip arthroplasty; TKA: total knee arthroplasty; PKA: partial knee arthroplasty; PJI: periprosthetic joint infection

Age	BMI	Surgery	Complication	Infection	Additional surgeries	Did the patient endorse receiving nutrition advice preoperatively?	Did the patient endorse following the nutrition advice preoperatively?
74	32.4	Left TKA	Temporary peroneal nerve palsy that resolved within three months without interventions	No	No	Yes	Yes
66	24.4	Left TKA	Postoperative superficial infection of cellulitis that resolved with antibiotics. No deep joint infection	No	No	Yes	No
69	23.4	Right TKA	Allergic reaction to the closing adhesive that resolved without interventions	No	No	Yes	Yes
66	27.7	Right PKA	Index PKA performed by an outside surgeon. The patient presented with a PJI, and the patient did not want care from the index surgeon	Yes	Yes: two-stage revision for infection	Yes	Yes
80	29.2	Left TKA	Postoperative PJI four months after index surgery after a traumatic wound while on vacation	Yes	Yes: two-stage revision for infection	Yes	Yes
63	25.9	Right TKA	Patient on immunosuppression for chemotherapy with postoperative PJI	Yes	Yes: two-stage revision for infection	Yes	Yes

Survey findings

Seventy-nine patients (54.1% of the total responses for this question (n = 146)) reported that their orthopedic surgeon was the first physician to counsel them about nutrition.

One hundred seventeen patients (80.1% of the total responses for this question (n = 146)) recalled receiving preoperative nutrition counseling from their surgeon.

One hundred eleven patients (76% of the total responses for this question (n = 146)) reported understanding how nutrition affects orthopedic care.

Fifty-seven patients (51.4% of the total responses for this question (n = 111)) did not believe they would have benefited from a referral to a nutritionist, 18 patients (16.2%) believed they would, and 36 patients (32.4%) were unsure.

Ninety-six patients (86.5% of the total responses for this question (n = 111)) would recommend their surgeon's nutrition counseling to others, 13 patients (11.7%) were unsure, and two patients (1.8%) would not.

One hundred fourteen patients (78.1% of the total responses for this question (n = 146)) indicated they wanted their surgeon to discuss nutrition with them, 17 (11.6%) were unsure, and 15 (10.3%) preferred not to have nutrition discussions with their surgeon.

One hundred twenty-seven patients (87% of the total responses for this question (n = 146)) reported that nutrition counseling by their surgeon positively impacted their care, 12 (8.2%) were unsure, and seven (4.8%) did not report a benefit.

Analysis

Since this was an exploratory analysis, we did not perform multivariable modeling or adjust for potential confounders, such as BMI, comorbidities, or socioeconomic status. Instead, we present descriptive results and a subgroup analysis to highlight potential associations that warrant further study.

Table 4 demonstrates the subgroup analysis, which showed that patients who followed a low-carbohydrate diet were slightly younger (mean: 69.6 years) than those who did not follow the diet (mean: 72.5 years; p = 0.054). No differences in complication rates were observed between the diet groups (p = 1.0).

**Table 4:** Comparison of participant age by adherence to a low-carbohydrate diet. A Welch two-sample t-test was used to compare the mean age between patients who reported following a low-carbohydrate diet and those who did not. Participants adhering to a low-carbohydrate diet were younger on average, though the difference was not statistically significant (p = 0.055).

Low-carbohydrate diet	N	Age (mean)	Age (standard deviation)	Difference in means (95% CI)	t-statistics	P-value
Yes	110	69.58426	8.476607	-2.93653 (-5.93138054, 0.05831923)	-1.9598	0.055449
No	34	72.52079	7.357238			

Exploratory analyses found no association between smoking status and complications (p = 1.0 for infection; p = 0.427 for overall complications). Alcohol consumption showed a nonsignificant trend toward higher infections with greater use (p = 0.84). Age was not significantly different between patients with and without complications (p = 0.6434). These are reported in Table 5.

**Table 5:** Categorical subgroup analyses to determine the difference in infection status based on smoking status, age, and alcohol use. Subgroup analyses were conducted for smoking status, age, and alcohol use to evaluate potential differences in infection rates. Fisher's exact test was used for categorical comparisons of smoking status, Welch's two-sample t-test for differences in age, and the Cochran-Armitage trend test for alcohol use.

Subgroup	N	95% confidence interval	Statistical test
Smoking status	148	(0.11, 12.28)	Fisher's exact test
Age	146	(-8.881206, 12.870667)	Welch two-sample t-test
Alcohol use	147	NA	Cochran-Armitage trend test

Table 6 demonstrates the subgroup analyses and exact cell counts for statistical tests.

**Table 6:** Subgroup analyses and exact cell counts for statistical tests. Low-carb: low-carbohydrate

Comparison	Exposure groups	Outcome category	Counts (n)	Statistical test	P-value
Smoking vs. infection	No = 99 (96 + 3); yes = 49 (47 + 2)	Infection (yes/no)	No infection = 143 (96 + 47); yes infection = 5 (3 + 2)	Fisher's exact	1.00
Smoking vs. any infection/complication	No = 99 (86 + 13); yes = 49 (45 + 4)	Any infection/complication (yes/no)	No = 131 (86 + 45); yes = 17 (13 + 4)	Fisher's exact	0.427
Low-carb diet vs. infection	No = 34 (33 + 1); yes = 112 (108 + 4)	Infection (yes/no)	No infection = 141 (33 + 108); yes infection = 5 (1 + 4)	Fisher's exact	1.00
Low-carb diet vs. any infection/complication	No = 34 (30 + 4); yes = 112 (99 + 13)	Any infection/complication (yes/no)	No = 129 (30 + 99); yes = 17 (4 + 13)	Fisher's exact	1.00
Age by low-carb diet	Yes (n = 112) (mean 69.6); no (n = 34) (mean 72.5)	-	-	Welch t-test	0.054
Age by infection	No infection (n = 143) (mean 70.37); infection (n = 5) (mean 68.37)	-	-	Welch t-test	0.643
Alcohol use vs. any infection/complication	Ordered 8-level alcohol variable	Any infection/complication (yes/no)	-	Cochran-Armitage trend	0.0661
Alcohol use vs. infection only	Ordered 8-level alcohol variable	Infection (yes/no)	-	Cochran-Armitage trend	0.84

Open-text responses (n = 95; 79 substantive comments) were analyzed for sentiment and revealed generally positive attitudes toward surgeon-led nutrition counseling, aligning with quantitative findings.

## Discussion

Optimizing patient health preoperatively for hip and knee arthroplasty remains a complex, multifactorial component of their care, with the role of nutrition gaining discussion [3]. There remain limitations in the understanding of the possible benefits and utility of nutrition counseling for arthroplasty patients by their treating orthopedic surgeon. In this study of a single-surgeon practice, a self-selected cohort of patients who responded to a survey retrospectively reported positive perceptions of nutrition discussions with their orthopedic surgeon. This study provides hypothesis-generating subjective, patient-centered perspectives that complement existing objective, outcome-driven studies. 

Guidelines, such as those from the ERAS Society, emphasize the importance of perioperative nutrition, but they also highlight variability in implementation across health systems [13]. In this context, our findings suggest that surgeon-led conversations may serve as an additional perceived benefit for patients, reinforcing multidisciplinary strategies that include dietitian or team-based interventions [14,15]. In a 2025 study by Galetti et al., they found that 94% of their patient respondents would alter their diet or nutrition goals if they understood the implications of nutrition on arthroplasty recovery [16]. Our survey demonstrated that 76% of participants expressed understanding of how nutrition affects their orthopedic care, which highlights the potential importance of these discussions. However, a patient's understanding of nutrition is limited by their health literacy, which is not addressed in this study.

Nutrition in orthopedic surgery is becoming more prevalent in discussions of patient care, but there remain limited studies or guidelines to help orthopedic surgeons navigate these patient conversations. Prior investigations have highlighted the importance of multidisciplinary approaches in considering weight loss and nutrition counseling before total joint arthroplasty [14]. However, delineating the role of an orthopedic surgeon in navigating nutrition counseling and making referrals to other specialists like nutritionists, bariatric surgeons, and weight-loss physicians remains unclear [14]. Prior literature has supported the utility of multidisciplinary teams for weight loss and nutrition management in orthopedic arthroplasty patients [14,15]. Yee et al. identified benefits for patients undergoing total joint arthroplasty when a perioperative dietitian-led intervention was introduced [15]. Many similar studies have focused on the benefits of outside resources with nutrition counseling prior to arthroplasty. However, it remains unclear how an orthopedic surgeon counseling on nutrition may affect patient outcomes or patient perceptions. Our retrospective study demonstrated that the majority of the participating patients perceived value from having their orthopedic surgeon discuss nutrition with them.

Limitations and future directions

This retrospective study surveyed patients over a 20-year span from a single private practice orthopedic surgeon, which limits generalizability to other practice settings. Future studies should assess different sites, such as academic or larger private practice groups, and other subspecialties beyond arthroplasty. The survey was distributed only via QR code without a paper alternative, which may have introduced response/selection bias and created barriers for less technologically comfortable patients. Although 144 of 152 respondents reported English as their primary language, the lack of translation services may have excluded non-English-speaking patients. Recall bias is also possible given the retrospective nature of the survey. Several questions had fewer responses toward the end of the 33-item survey, suggesting survey length may have been a barrier to completion. This study is ultimately limited by the subjective, unvalidated patient perceptions of this survey-based study.

No power analysis was performed, which may increase the risk of type II error. The small sample size (n = 152) for a 20-year period and the low number of complications (n = 6) limit the ability to detect associations, especially in a subgroup analysis. Chart reviewers were not blinded to survey responses, introducing potential abstraction bias. Outcomes were based on subjective patient perceptions rather than validated patient-reported outcome measures (PROMs) or objective measures, and compliance with nutrition recommendations was not assessed. This leads to high potential for coercion and social desirability bias as patients may feel pressured to report positive feedback about their treating surgeon's advice. A surgeon-patient relationship is a likely confounder that could artificially inflate positive perceptions. 

We did not adjust for key confounders, such as BMI, comorbidities, or socioeconomic status, limiting causal inference. Exploratory comparisons without multivariable modeling or multiplicity correction (including p < 0.1 thresholds) increase the risk of type I error. Collectively, these factors underscore that results should be considered hypothesis-generating. In addition, our complication data were descriptive and not linked statistically to counseling, preventing causal conclusions. 

There are also many different methods of nutrition counseling with variability in recommendations, leading to difficulty providing standardized evidence-based recommendations. The single operating surgeon in this study focused on general nutrition education and dietary modifications for a low-carbohydrate diet with increased protein intake, so there were no comparisons in dietary patterns of the patients with outcomes. This study was not able to assess patient adherence with the nutrition recommendations. Therefore, this study is not able to recommend any specific nutrition counseling guidelines for arthroplasty patients, which would be of benefit for future investigations. Due to the many limitations of this study (e.g., retrospective design, selection bias, recall bias, small sample size, lack of confounding control, and subjective outcomes), the results cannot support any patient-reported benefits or suggest any definitive associations.

Despite these limitations, this study provides hypothesis-generating insight into patient perspectives on surgeon-led nutrition counseling. Our findings suggest that patients valued discussions about nutrition with their orthopedic surgeon, even within the exploratory framework, but no associations can be made due to the study's limitations. Whereas most prior work has emphasized structured, dietitian-led interventions, this study highlights the potential complementary role of surgeons in initiating these conversations. These observations may inform how orthopedic teams integrate nutrition counseling into preoperative planning, particularly in resource-limited settings where access to dietitians may be constrained. This study highlights the need for future studies, such as a prospective multicenter study with a validated questionnaire, improved power, adjustment for confounders, and improvement in assessing objective outcomes, to assess the impact of nutrition counseling by orthopedic surgeons on arthroplasty patient outcomes.

## Conclusions

In a single-surgeon practice, a self-selected cohort of patients who responded to a digital survey retrospectively reported positive perceptions of nutrition discussions with their orthopedic surgeon. This study cannot determine whether these discussions provided any clinical benefit, influenced behavior, or are superior to other methods of delivering nutritional information. Although limited by its retrospective, single-surgeon design and reliance on patient self-reporting, this study suggests the potential for surgeon-delivered counseling to complement multidisciplinary strategies. This study only provides preliminary, hypothesis-generating data on patient attitudes, not evidence of efficacy or benefit. Therefore, the findings indicate patient receptivity to this topic, which justifies future investigation using prospective, controlled designs with objective outcome measures.

## Figures and Tables

**Figure 1:**
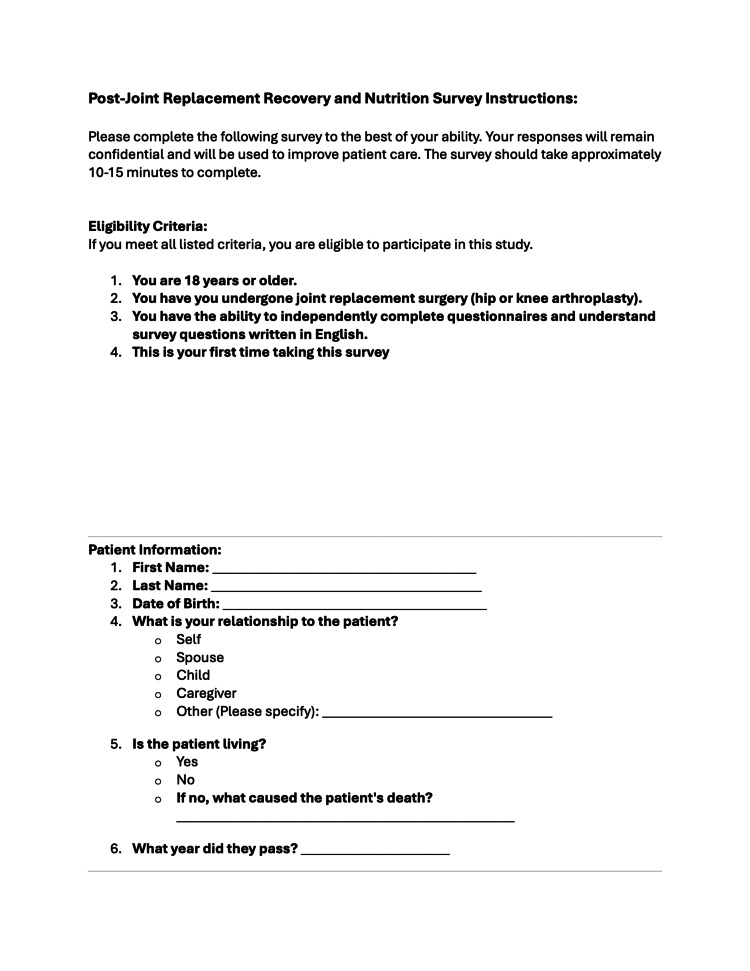
Survey: page 1

**Figure 2:**
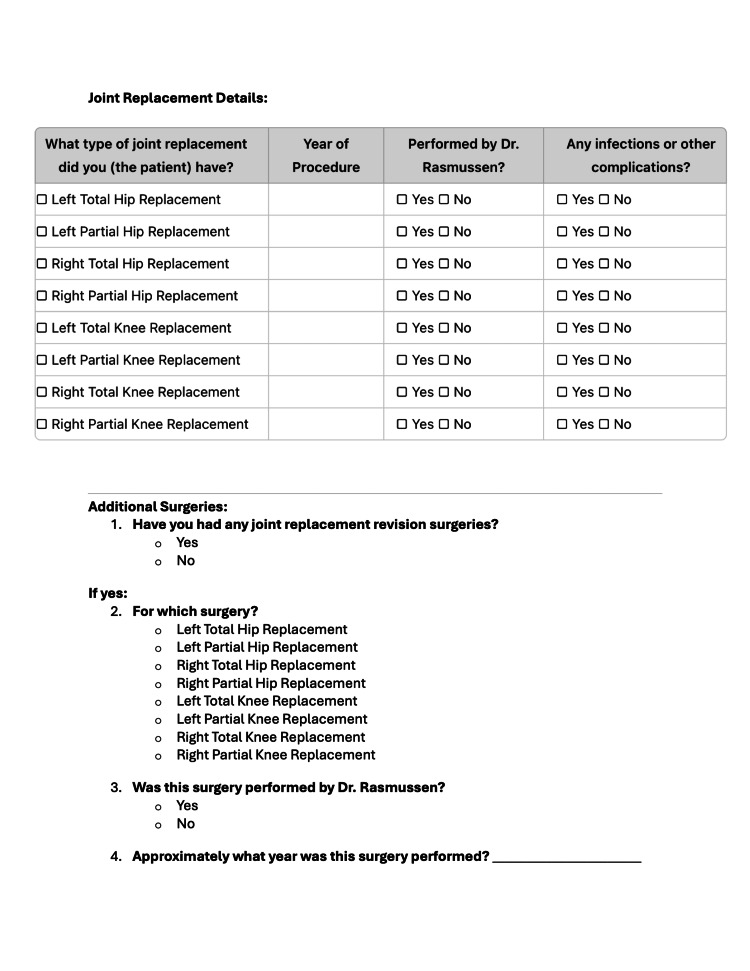
Survey: page 2

**Figure 3:**
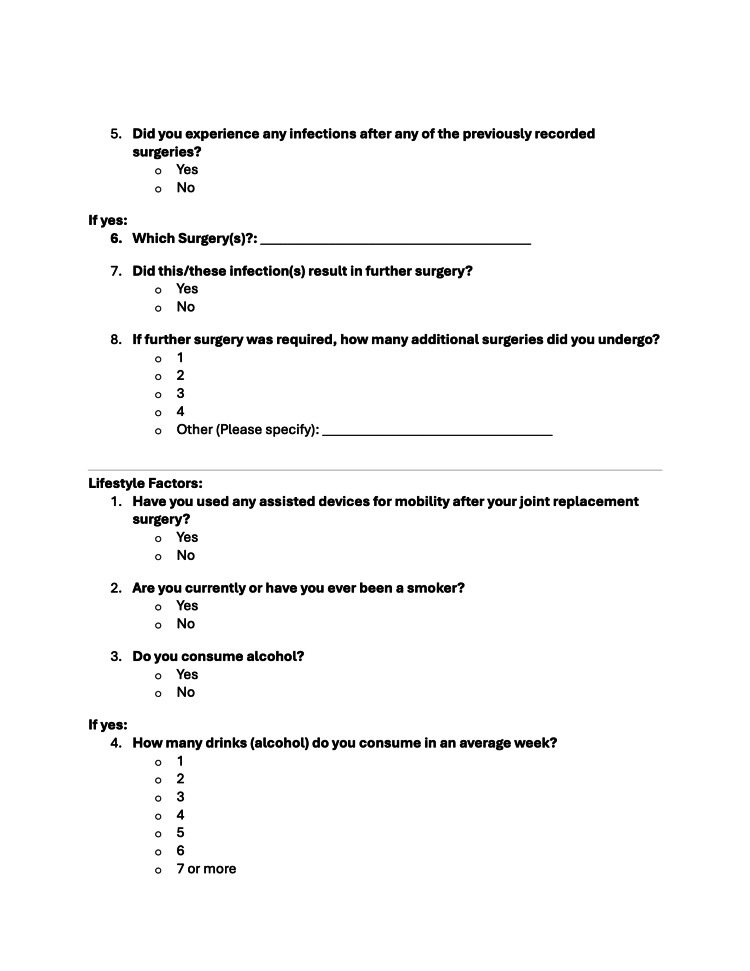
Survey: page 3

**Figure 4:**
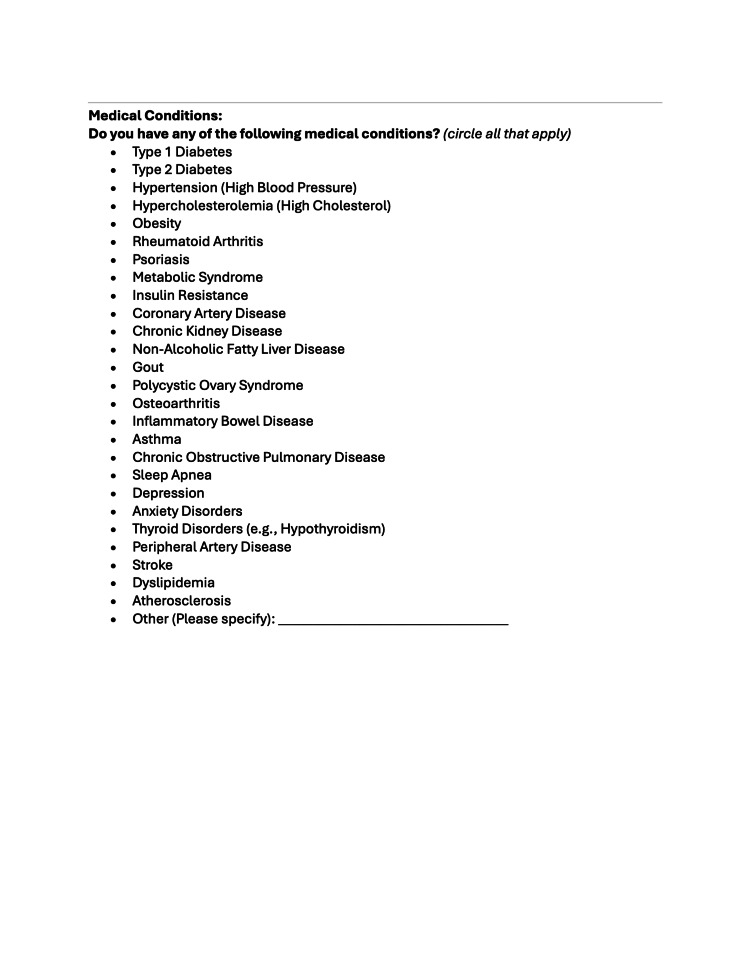
Survey: page 4

**Figure 5:**
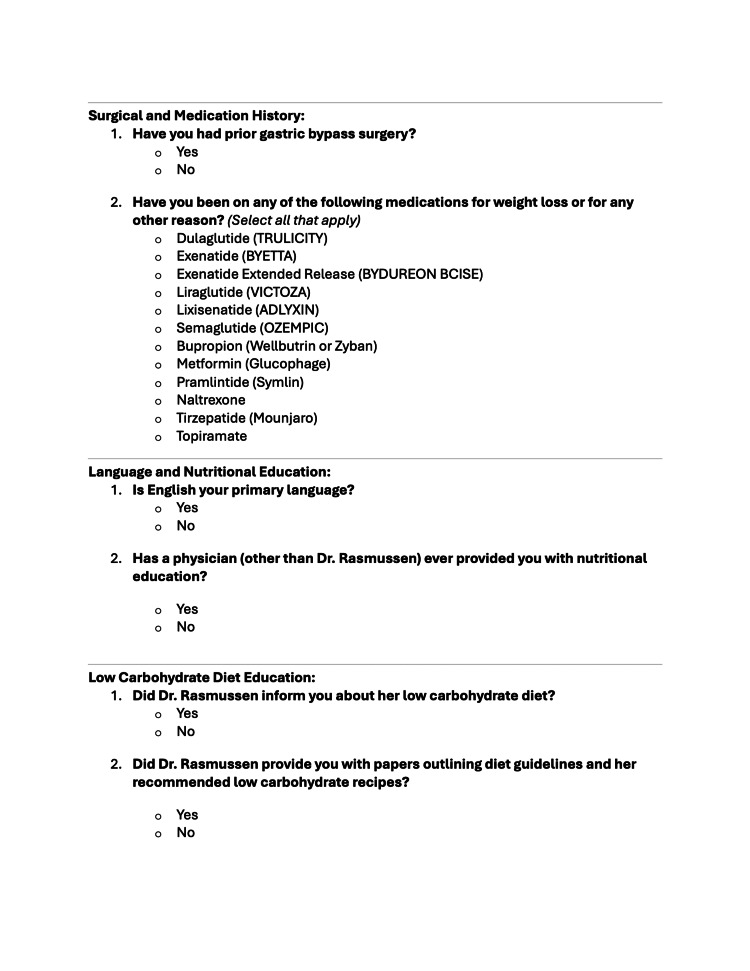
Survey: page 5

**Figure 6:**
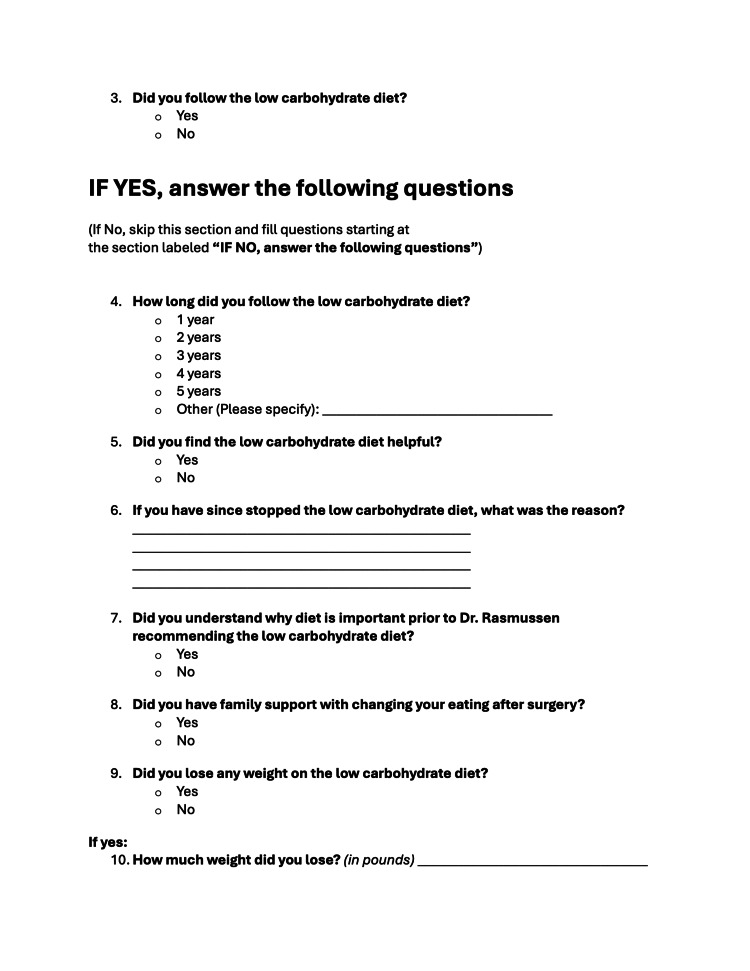
Survey: page 6

**Figure 7:**
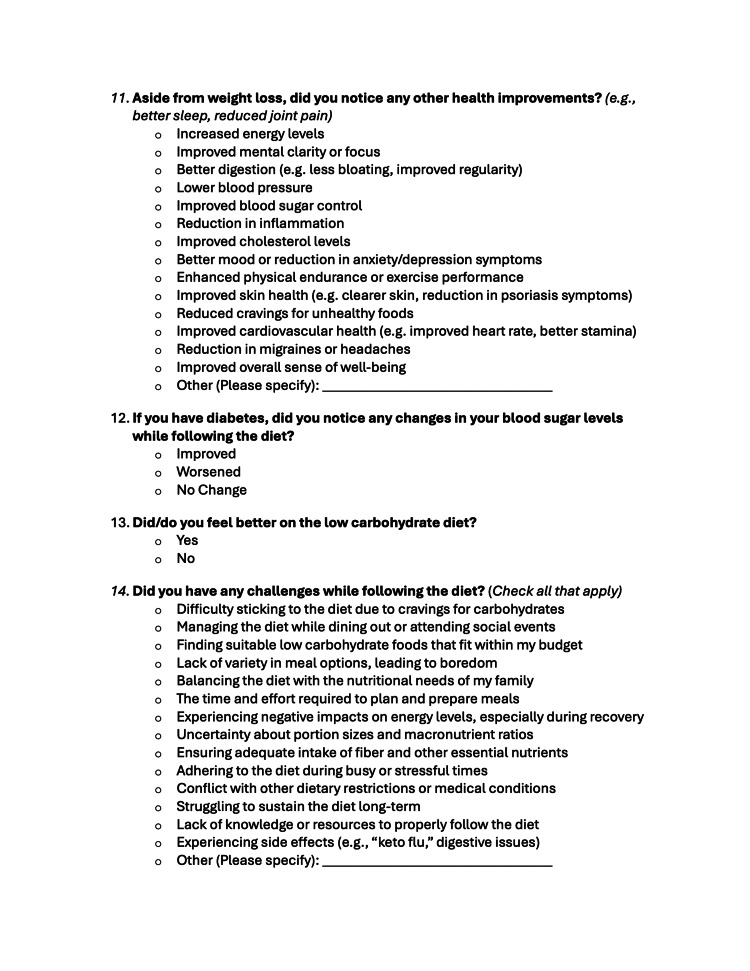
Survey: page 7

**Figure 8:**
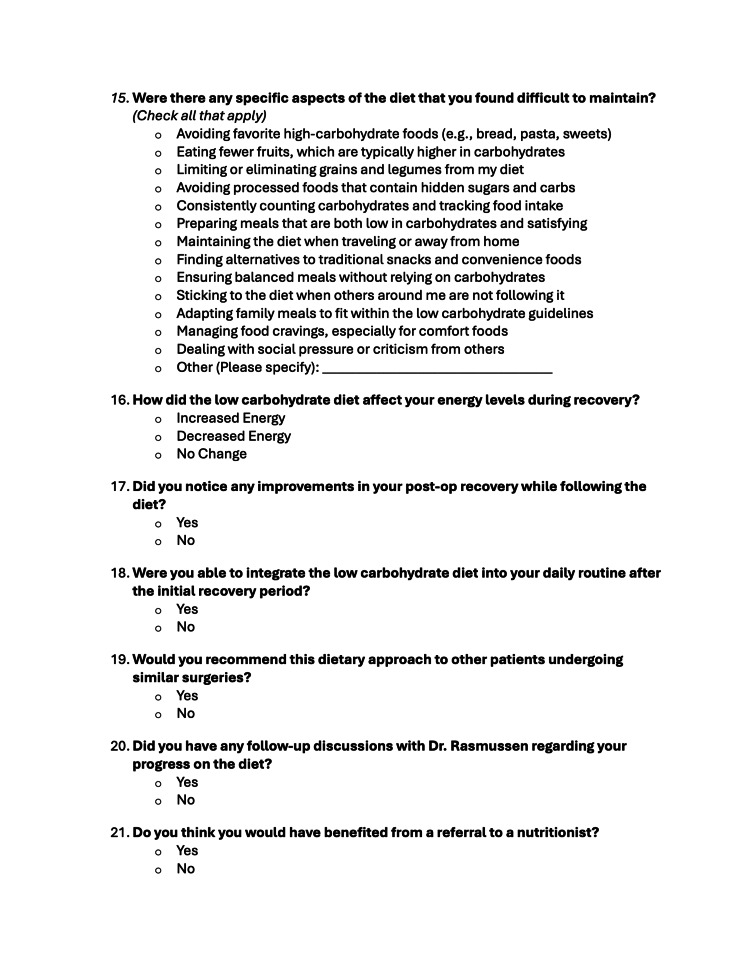
Survey: page 8

**Figure 9:**
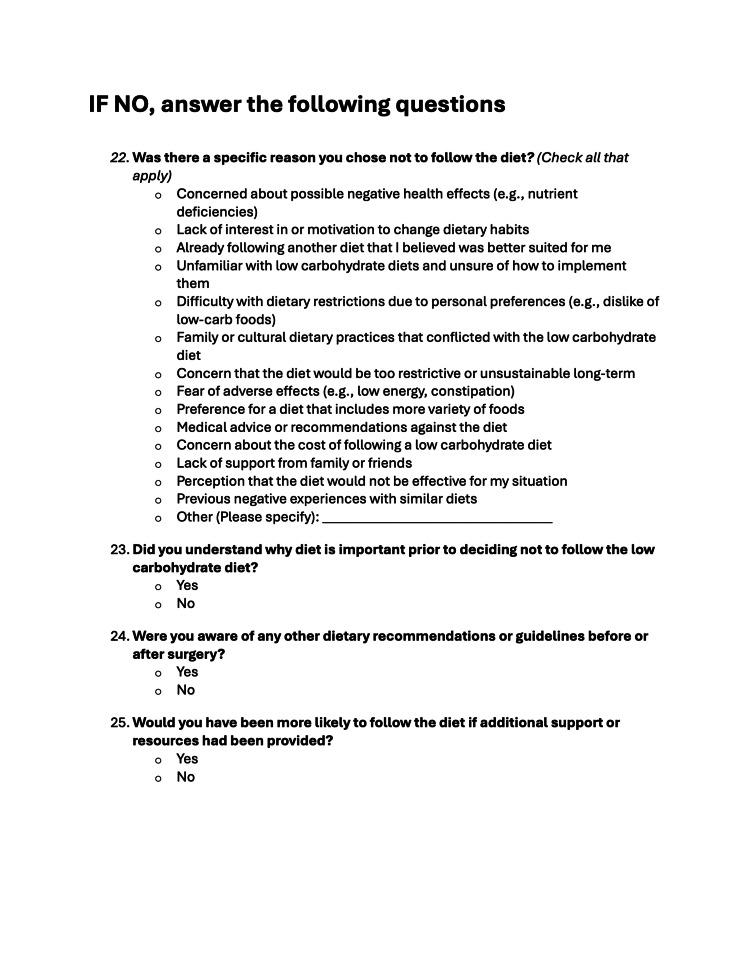
Survey: page 9

**Figure 10:**
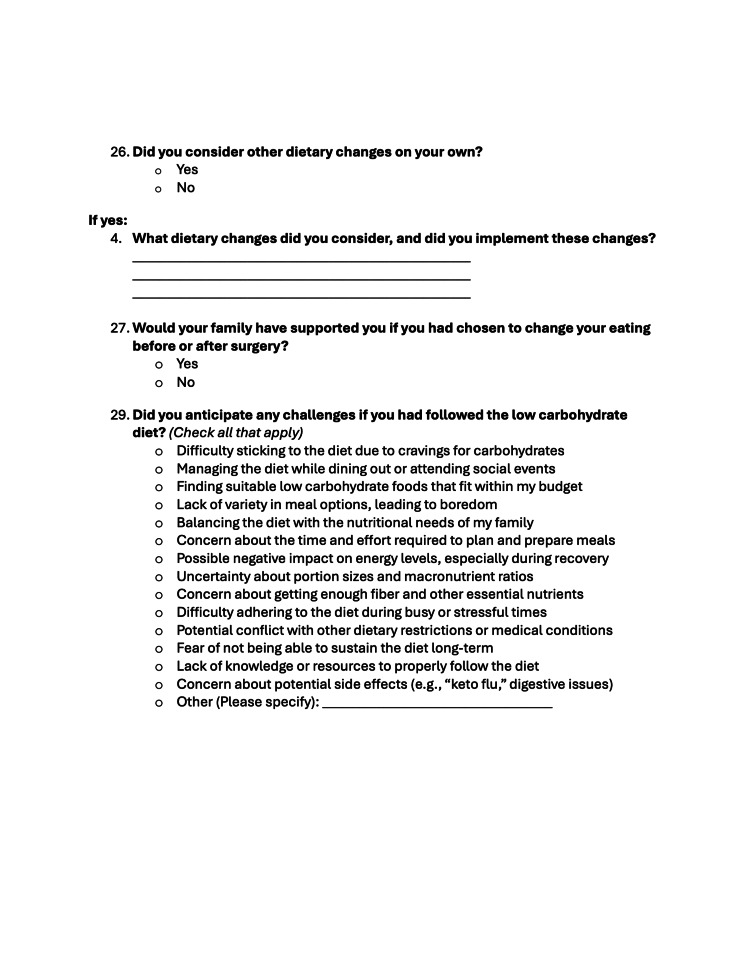
Survey: page 10

**Figure 11:**
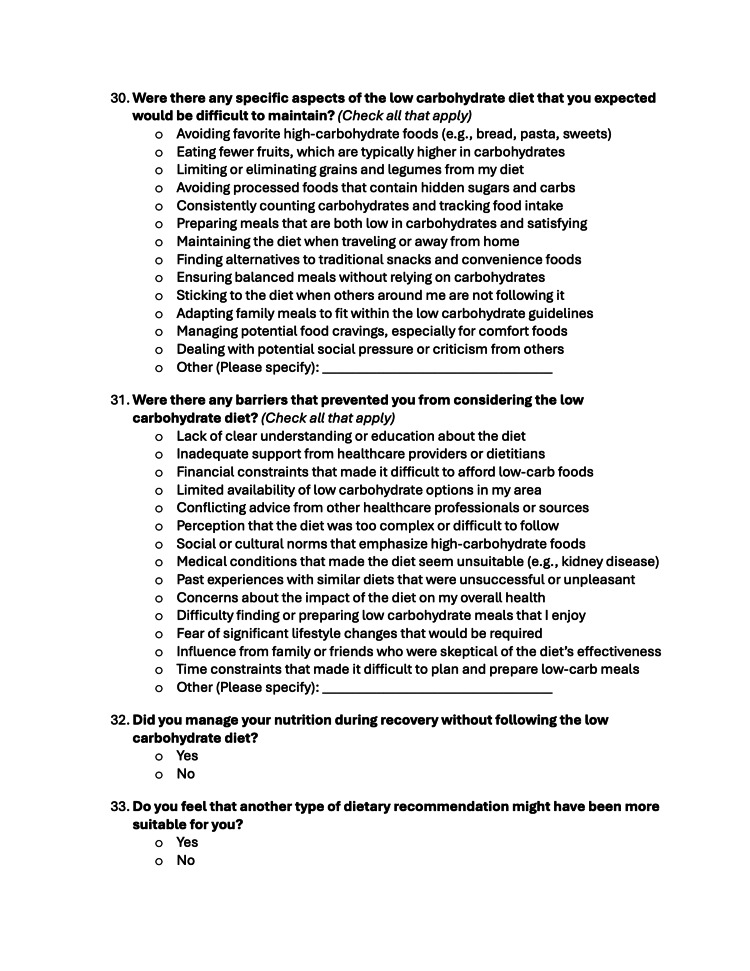
Survey: page 11
